# Modeling Tumor Microenvironment Complexity In Vitro: Spheroids as Physiologically Relevant Tumor Models and Strategies for Their Analysis

**DOI:** 10.3390/cells14100732

**Published:** 2025-05-17

**Authors:** Shrey Shah, Gerard G. M. D’Souza

**Affiliations:** 1 Department of Pharmaceutical Sciences, Massachusetts College of Pharmacy and Health Sciences, Boston, MA 02115, USA; gerard.dsouza@mcphs.edu; 2 Atom Bioworks Inc., Cary, NC 27513, USA

**Keywords:** tumor microenvironment, multicellular tumor spheroids, 3D cell culture analysis

## Abstract

Drug delivery to solid tumors is challenged by multiple physiological barriers arising from the tumor microenvironment, including dense extracellular matrix, cellular heterogeneity, hypoxic gradients, and elevated interstitial fluid pressure. These features hinder the uniform distribution and accumulation of therapeutics, reducing treatment efficacy. Despite their widespread use, conventional two-dimensional monolayer cultures fail to reproduce these complexities, contributing to the poor translational predictability of many preclinical candidates. Three-dimensional multicellular tumor spheroids have emerged as more representative in vitro models that capture essential features of tumor architecture, stromal interactions, and microenvironmental resistance mechanisms. Spheroids exhibit spatially organized regions of proliferation, quiescence, and hypoxia, and can incorporate non-tumor cells to mimic tumor–stroma crosstalk. Advances in spheroid analysis now enable detailed evaluation of drug penetration, cellular migration, cytotoxic response, and molecular gradients using techniques such as optical and confocal imaging, large-particle flow cytometry, biochemical viability assays, and microfluidic integration. By combining physiological relevance with analytical accessibility, spheroid models support mechanistic studies of drug transport and efficacy under tumor-like conditions. Their adoption into routine preclinical workflows has the potential to improve translational accuracy while reducing reliance on animal models.

## 1. Introduction

The therapeutic management of solid tumors remains one of the most challenging areas in oncology due to the complex physiological and structural barriers that limit drug efficacy [1,2]. Many anticancer agents fail to achieve sufficient intratumoral concentrations or exhibit poor distribution within tumor tissues, resulting in incomplete eradication of malignant cells and the development of therapeutic resistance [3]. These challenges are further amplified by the heterogeneous composition of solid tumors, which includes not only cancer cells but also a diverse population of stromal and immune cells present within a dense extracellular matrix (ECM) [4,5]. Together, these components create a tumor microenvironment (TME) that significantly influences drug penetration, retention, and therapeutic response [1,3,4]. Despite these complexities, preclinical drug screening has primarily relied on monolayer cultures that lack the architectural and biochemical features of in vivo tumors [6]. While these models are convenient and scalable, they fail to capture essential characteristics such as spatial cell–cell interactions, oxygen and nutrient gradients, and ECM-mediated diffusion resistance—factors that critically influence drug behavior in solid tumors [6,7]. Beyond these structural and biochemical limitations, the phenotypic state of cancer cells is also profoundly influenced by their dimensional context [8,9]. When cultured in 3D systems, tumor cells often exhibit altered expression of key molecular markers associated with proliferation, apoptosis, drug resistance, and differentiation, compared to their 2D counterparts [10]. These changes can significantly impact how cells respond to therapeutic agents and contribute to discrepancies between in vitro predictions and in vivo outcomes [10]. Together, this disconnect between conventional monolayer models and the complexity of the in vivo tumor environment contributes to the high rate of failure observed during the transition from preclinical studies to clinical development [11,12,13].

Multicellular tumor spheroids have emerged as a promising platform to bridge this translational gap [14,15]. By mimicking the hierarchical structure, proliferation gradients, and stromal interactions found in native tumor tissues, spheroids provide a more physiologically relevant context for evaluating anticancer agents and delivery systems [16,17]. This review explores the utility of spheroid models in modeling the tumor microenvironment, assessing drug penetration and efficacy, and enabling high-content analytical techniques for characterizing spatial and functional heterogeneity. The discussion begins with an overview of the tumor microenvironment and its impact on therapeutic response.

## 2. Understanding the Tumor Microenvironment

**Figure 1:**
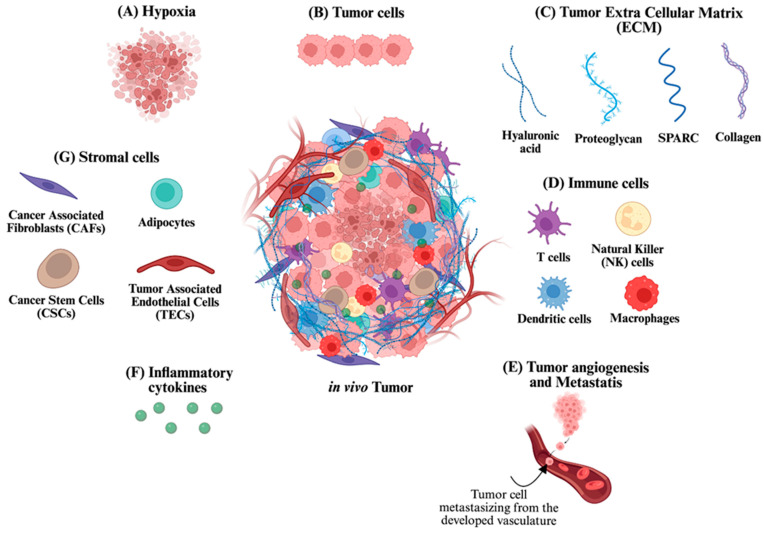
**Schematic representation of the tumor microenvironment (TME) and its key components.** (**A**) Hypoxia: Resulting from disorganized and inefficient vasculature, leading to oxygen deprivation in the tumor core and promoting survival adaptations and therapy resistance. (**B**) Tumor cells: The central malignant population that interacts with and reshapes the surrounding microenvironment. (**C**) Extracellular matrix (ECM): A structurally and biochemically complex network composed of fibrous proteins (e.g., collagen), structural glycoproteins (such as SPARC), proteoglycans, and glycosaminoglycans, like hyaluronic acid. These components not only provide mechanical support but also modulate cellular adhesion, migration, and signal transduction. (**D**) Immune cells: Includes T cells, B cells, macrophages, dendritic cells, and NK cells that exhibit both anti-tumor and immunosuppressive roles. (**E**) Angiogenesis: Aberrant formation of blood vessels induced by tumor and stromal signaling; supports oxygen/nutrient delivery and facilitates metastasis via increased vascular permeability. (**F**) Inflammatory cytokines: Soluble mediators such as IL-6, TNF-α, TGF-β, and VEGF that promote chronic inflammation, immune suppression, angiogenesis, and stromal activation. (**G**) Stromal components: Includes cancer-associated fibroblasts, adipocytes, mesenchymal stem cells, tumor associated endothelial cells that regulate ECM remodeling, metabolic support, and immune modulation.

Earlier theories of tumor biology proposed that tumor initiation results from genetic alterations within a single cell [18,19]. This mutation drives abnormal proliferation, leading to the formation of a solid cell mass. Tumor progression was subsequently attributed to the acquisition of additional mutations, generating malignant clones capable of invasion, extravasation, and metastasis [18,19]. However, this view has evolved substantially. It is now widely accepted that tumor initiation and progression are not governed solely by genetic mutations but are significantly influenced by the dynamic interplay between tumor cells and their surrounding microenvironment. This surrounding landscape—referred to as the tumor microenvironment (TME)—comprises a complex network of cellular, acellular, and immune components [5,20,21]. The cellular compartment includes both tumor cells and stromal cells, the latter primarily composed of fibroblasts, endothelial cells, and adipocytes. The acellular component is defined by the ECM, a structural meshwork composed of cross-linked protein molecules such as laminin, fibronectin, collagen, and hyaluronan (Figure 1). These components are not passive bystanders; rather, they actively communicate through biochemical and biomechanical signaling to influence tumor behavior [5,22,23]. Importantly, this crosstalk is bidirectional: while tumor cells reshape the microenvironment to support growth and metastasis, stromal and ECM elements also exert selective pressures that drive tumor adaptation and evolution [24,25,26].

In the past, due to the limited understanding of the roles played by stromal and acellular elements, anti-cancer therapies were designed almost exclusively to target tumor cells. This narrow focus overlooked the significant contributions of non-tumor components in modulating tumor behavior. As evidence accumulated showing that stromal cells and ECM significantly influence tumor development, resistance, and metastasis, the therapeutic paradigm shifted. There is now a growing appreciation for the need to target both tumor cells and their supportive microenvironment [22,23]. A deeper understanding of the TME not only expands the pool of potential therapeutic targets but also enables the development of more predictive and physiologically relevant preclinical models. The subsequent sections will provide a concise overview of the distinct role of stromal cells, immune components, and the ECM in tumor development and progression.

### 2.1. Fibroblasts and Their Role in Tumor Progression

Among the various stromal cells within the TME, fibroblasts constitute the dominant cellular mass [27,28,29,30]. In normal physiology, fibroblasts are essential for maintaining tissue integrity and play pivotal roles in inflammation, wound healing, and fibrosis [29]. Early research into their role in melanoma progression revealed that fibroblasts exert opposing effects depending on the malignancy stage of tumor cells [27]. In early tumorigenesis, fibroblasts appeared to inhibit tumor growth, whereas in advanced stages, they promoted tumor expansion [27]. This dual behavior was attributed to the secretion of distinct soluble factors, including both inhibitory and stimulatory growth mediators [27]. Importantly, the functional outcome of these signals is not determined solely by fibroblast activity but is strongly shaped by the phenotypic state of the tumor cells. Factors such as epithelial-to-mesenchymal transition (EMT), oncogene activation, metabolic reprogramming, and altered expression of growth factor receptors can modulate how tumor cells perceive and respond to fibroblast-derived cues [28,31,32]. For example, mesenchymal-like cancer cells often exhibit increased motility and invasiveness in response to fibroblast-secreted TGF-β or IL-6, while more epithelial-like cells may remain growth-arrested or less responsive. Thus, the fibroblast–tumor cell interaction is not only context-dependent but also bidirectionally shaped by the evolving molecular and functional landscape of the tumor itself [33,34].

With the advancement of tumor biology, it became clear that tumor cells actively reprogram surrounding fibroblasts into a modified state termed cancer-associated fibroblasts (CAFs) [5,19,35,36]. These CAFs are characterized by the expression of specific markers, such as desmin and fibroblast activation protein, and are now recognized as key contributors to ECM remodeling, angiogenesis, and metastasis [36,37,38,39,40]. In a preclinical model of tumor progression, the presence of fibroblasts enhanced the survival of circulating tumor cells, facilitating their colonization at distant sites following primary tumor dissemination [41]. Notably, depletion of CAFs significantly reduced metastatic burden and extended survival following tumor resection [41].

In addition to structural remodeling, CAFs serve as metabolic source of nutrients for cancer cells. Tumor cells induce oxidative stress in adjacent fibroblasts, triggering the production of reactive oxygen species (ROS) [42,43,44]. This ROS burden initiates autophagic processes in fibroblasts, leading to the generation of energy-rich metabolites such as ketones, lactate, and pyruvate. These metabolites are shuttled to adjacent tumor cells and support mitochondrial oxidative phosphorylation, thereby sustaining tumor cell growth and bioenergetic demands [42,43,44,45].

### 2.2. Endothelial Cells and Tumor Angiogenesis

Endothelial cells play a critical role in facilitating tumor vascularization through the process of angiogenesis, thereby enabling tumor cells to meet increasing metabolic demands [46]. This neovascularization represents a rate-limiting step in tumor progression [47]. Small tumors (less than ~1–2 mm^3^ in volume) are generally avascular and rely on diffusion from nearby vessels for oxygen and nutrients [48,49]. As the tumor expands beyond this size, the limited diffusion capacity results in the formation of hypoxic regions, which drive tumor dormancy [50,51]. This hypoxic stress acts as a potent inducer of angiogenic signaling, upregulating key factors such as vascular endothelial growth factor (VEGF) [51]. When VEGF binds to its cognate receptors on endothelial cells, it promotes their activation, proliferation, and directional migration toward the hypoxic tumor mass. This is followed by vascular sprouting and lumen formation, collectively resulting in the generation of a new, albeit abnormal, vascular network within the tumor [49,52]. The newly formed vasculature supports continued tumor proliferation by restoring oxygen and nutrient supply and also facilitates metastasis through enhanced intravasation of tumor cells into the circulation [53,54].

Unlike normal vasculature, tumor-associated blood vessels are structurally aberrant [54]. They display irregular diameters, lack the hierarchical architecture typical of the arterial-arteriolar-capillary network, and exhibit increased permeability and fragility [55,56]. These abnormalities result in heterogeneous blood flow, with some vessels being poorly perfused or entirely non-functional, while others demonstrate turbulent or reversed flow patterns. The resulting hemodynamic instability contributes to regional hypoxia and impedes the uniform distribution of therapeutics, thereby reducing drug delivery efficacy and promoting resistance [54].

### 2.3. Adipocytes: Metabolic Modulators in the TME

Adipocytes constitute a significant stromal component in the TME of several malignancies, including breast, ovarian, and colorectal cancers [57,58]. Within the TME, these adipocytes exhibit a markedly altered phenotype compared to their counterparts in normal tissue [59]. Morphological changes include reduced cell size, expanded interstitial spacing, and a pronounced decrease in intracellular lipid content [59]. These alterations reflect a shift toward a metabolically active state, wherein adipocytes undergo lipolysis to release free fatty acids (FFAs). These FFAs are readily taken up by adjacent tumor cells and utilized for multiple oncogenic processes, including ATP generation via β-oxidation, synthesis of membrane phospholipids, and production of lipid-based signaling molecules that regulate proliferation and survival. Thus, adipocytes function not only as structural components of the TME but also as metabolic contributors to tumor progression [60].

Mechanistic studies in breast cancer models have demonstrated that mature adipocytes co-cultured with tumor cells acquire an activated phenotype, characterized by upregulation of matrix metalloproteinases (e.g., MMP-11) and pro-inflammatory cytokines, such as interleukin (IL)-6 and IL-1β [61]. These molecular changes facilitate ECM remodeling, enhance tumor cell invasion, and promote EMT [61]. Furthermore, in vivo experiments revealed that co-injection of activated adipocytes with breast cancer cells significantly increased pulmonary metastases compared to injections of tumor cells alone. Collectively, these findings establish adipocytes as dynamic modulators of tumor metabolism, invasion, and dissemination [61]. All these findings suggest the importance of adipocytes in tumor progression.

### 2.4. Immune Cell Dynamics in Tumor Progression and Resistance

Immune cells, although not classified as stromal cells, are key infiltrative components of the TME and play multifaceted roles in regulating tumor dynamics. Their functional impact is context-dependent and can be dichotomized into anti-tumorigenic and pro-tumorigenic subsets [46,62]. Anti-tumor immune cells include cytotoxic CD8⁺ T lymphocytes, Natural Killer (NK) cells, M1-polarized macrophages, and dendritic cells. These cells mediate tumor suppression through mechanisms such as antigen presentation, secretion of pro-inflammatory cytokines (e.g., interferon-γ, TNFα), production of ROS, and direct cytotoxic killing of malignant cells [62,63]. Conversely, pro-tumor immune populations—such as regulatory T cells (Tregs), myeloid-derived suppressor cells (MDSCs), and M2-polarized macrophages—are involved in suppressing immune surveillance, promoting angiogenesis, and facilitating metastasis [62,64,65].

Among these, tumor-associated macrophages (TAMs) are frequently the most abundant immune subset within solid tumors [46]. Hypoxia, a hallmark of advanced malignancies, plays a pivotal role in TAM recruitment. Hypoxic tumor cells secrete chemokines (e.g., CCL2), hypoxia-inducible factors (HIF-1α, HIF-2α), and endothelin-2, which direct macrophage chemotaxis into the tumor core [66]. Once within the TME, macrophages display remarkable phenotypic plasticity [67,68]. This ability of macrophages is also known as macrophage polarization [67,68]. This polarization is stimulus-dependent: classically activated (M1) macrophages adopt a pro-inflammatory, anti-tumor phenotype, while alternatively activated (M2) macrophages acquire an immunosuppressive, pro-tumor phenotype [40,69]. Tumor-derived cues generally favor M2 polarization, resulting in the secretion of cytokines such as IL-4, IL-6, IL-1β, and TNFα. These cytokines support tumor growth by enhancing cellular proliferation, remodeling the ECM, and inhibiting effector T-cell responses. Thus, TAMs serve as both immunological suppressors and facilitators of malignant progression [70,71].

### 2.5. The Extracellular Matrix in TME Remodeling

The extracellular matrix (ECM), a non-cellular component of the TME, is a highly dynamic and structurally complex scaffold that plays a critical role in regulating tumor progression and therapeutic response [72,73,74]. The ECM is composed of a variety of cross-linked protein macromolecules—including collagen, fibronectin, laminin, and hyaluronan—synthesized primarily by CAFs and tumor cells [75,76].

This biochemical framework imparts key biophysical properties to the tumor tissue, including stiffness, porosity, insolubility, and molecular density [5,77,78,79]. These characteristics serve as biomechanical cues that modulate diverse cellular processes, such as migration, proliferation, polarity maintenance, and lineage specification [25,80]. Importantly, ECM composition is not static; it undergoes continuous remodeling through enzymatic degradation, de novo synthesis, and reorganization, all of which are tightly regulated by the cellular constituents of the TME [79,81]. This dynamic reciprocity between tumor cells and ECM components forms the basis of a feedback loop wherein changes in matrix architecture influence cell behavior and vice versa. For example, ECM remodeling can promote asymmetric division in cancer stem cells, disrupt tissue polarity, or facilitate directional migration during invasion [79,82]. In addition to its structural and signaling roles, the ECM actively contributes to tumor angiogenesis [83,84]. Proteolytic fragments of collagen types IV and XVIII—such as endostatin, canstatin, arresten, and tumstatin—modulate the activity of pro- and anti-angiogenic factors, including VEGF, thereby influencing endothelial cell behavior and guiding neovascular sprouting [85].

From a therapeutic standpoint, the ECM represents a formidable barrier to drug delivery. Its dense, cross-linked architecture hinders the diffusion and penetration of therapeutic agents [86]. In advanced tumors, excessive ECM deposition leads to mechanical compression of adjacent blood and lymphatic vessels, resulting in elevated interstitial fluid pressure (IFP) [87]. This elevated IFP diminishes convective transport, reduces vascular perfusion, and severely impairs drug accumulation in the tumor core [88,89]. Additionally, increased ECM stiffness and compactness restrict intercellular space, further compromising the efficacy of therapeutic delivery [89,90]. However, beyond acting as a physical barrier, ECM stiffness also triggers mechanotransduction pathways that profoundly influence tumor cell behavior [91,92]. Mechanical cues generated by a stiffened matrix are sensed through integrin-based focal adhesions and transmitted via cytoskeletal tension to activate intracellular signaling cascades, including Yes-associated protein/Transcriptional coactivator with PDZ-binding motif (YAP/TAZ), Focal Adhesion Kinase (FAK), and Mitogen-activate Protein Kinase (MAPK) pathways [93,94,95]. These signals modulate gene expression profiles associated with proliferation, EMT, and drug resistance [95,96]. This mechanosensitive response is particularly significant in desmoplastic tumors, such as pancreatic and breast cancers, where excessive collagen deposition and cross-linking create a dense fibrotic stroma [97]. In these settings, increased stiffness not only impedes drug penetration but also fosters an aggressive tumor phenotype and enhances resistance to chemotherapeutic agents [92,98]. Collectively, these biophysical and mechanobiological properties of the ECM represent critical barriers to effective treatment and are important targets for therapeutic modulation.

## 3. TME Features Impeding Drug Delivery

While the structural and cellular complexity of the TME has been extensively characterized, its direct consequences on therapeutic delivery remain a central challenge in cancer treatment. Dense ECM, cellular heterogeneity, and irregular vasculature create a diffusion-limited environment in which drugs fail to penetrate uniformly. Additionally, high interstitial fluid pressure and poor lymphatic drainage result in inefficient convective transport, further restricting access to tumor cores. These barriers are dynamic and spatially heterogeneous, factors that conventional 2D cultures and poorly structured models fail to recapitulate. Thus, a mechanistic understanding of how the TME impairs drug delivery is essential to design effective therapeutics and evaluate them in models that reflect in vivo transport limitations.

### 3.1. 3D Architecture

The three-dimensional (3D) architecture of solid tumors presents significant challenges to the effective penetration and distribution of therapeutic agents [99,100]. Drug efficacy within a tumor mass is frequently observed to be inversely correlated with the distance from the tumor periphery [101]. This spatial limitation is primarily attributed to architectural and vascular irregularities that create steep gradients in oxygen, nutrient availability, and pH [102].

One hallmark of this architecture is the development of hypoxic regions, typically located in the tumor core. These arise due to the disparity between the rapid proliferation of tumor cells and the slower proliferation and disorganized arrangement of endothelial cells responsible for vascularization [103,104]. This increased proliferation rate forces the nearby blood and lymphatic vessels apart [105]. The uncoordinated expansion of tumor cells physically displaces adjacent blood and lymphatic vessels, further impairing vascular function and exacerbating hypoxia [105]. These central regions are poorly perfused and metabolically stressed, whereas peripheral tumor cells—being in closer proximity to functional vasculature—exhibit higher proliferative indices [106].

Under hypoxic conditions, central tumor cells rely heavily on anaerobic metabolism, resulting in the accumulation of lactic acid and carbonic acid, which collectively reduce the extracellular pH [107,108]. These localized pH differences, in combination with proliferation gradients, significantly impact drug pharmacodynamics. For instance, the efficacy of chemotherapeutic agents that function through oxygen-dependent ROS generation—such as doxorubicin, cisplatin, and imexon—is markedly diminished in hypoxic regions due to insufficient oxygen availability [109,110,111,112]. Similarly, agents like paclitaxel, which preferentially target rapidly dividing cells, exhibit reduced cytotoxicity in the low-proliferation zones of tumor cores [99,113]. Additionally, the acidic extracellular environment alters the ionization state of many chemotherapeutic agents [114,115,116]. Because a significant number of anti-cancer drugs (e.g., melphalan, busulfan, carboplatin, and cisplatin) are weak bases, their increased ionization under acidic conditions limits their transmembrane diffusion and cellular uptake, ultimately reducing therapeutic efficacy [117,118].

### 3.2. Cellular Heterogeneity and Tumor Stroma

Tumors are inherently heterogeneous, consisting of multiple cancer cell subpopulations as well as diverse stromal constituents. Two prevailing hypotheses explain this heterogeneity: (1) clonal evolution, where genetic and epigenetic changes in initially homogenous tumor cells give rise to distinct subclones over time, and (2) the presence of cancer stem cells (CSCs) from the outset, which generate hierarchically organized progenies with varied phenotypes [119,120,121]. Both models contribute to drug resistance through different but overlapping mechanisms [119,120]. CSCs, in particular, exhibit intrinsic resistance to chemotherapy via overexpression of ATP-binding cassette (ABC) drug transporters, enhanced DNA repair capabilities, upregulation of anti-apoptotic proteins, and altered expression of drug metabolism-associated markers [122,123,124]. For example, in estrogen receptor-alpha (ERα)-positive breast tumors, CSCs have been shown to express significantly lower levels of ERα, a phenotype associated with resistance to endocrine therapy. This resistance has been mechanistically linked to Phosphoinositide 3-kinase (PI3K) pathway hyperactivation, which promotes proliferation and impairs responsiveness to hormonal agents [125]. Notably, CSCs do not function in isolation. Their survival and resistance characteristics are reinforced by neighboring non-CSC tumor cells that help maintain a specialized microenvironment or “stem cell niche” [126,127]. In ERα-positive breast cancer, for instance, CSCs may rely on ERα-positive non-CSC populations for paracrine signaling support, reinforcing drug resistance and promoting tumor persistence [128,129]. In addition to these models, EMT plays a critical role in driving phenotypic diversity in solid tumors. Through EMT, epithelial cancer cells undergo a dynamic reprogramming process that suppresses epithelial markers, such as E-cadherin, while upregulating mesenchymal markers, like vimentin and N-cadherin [130,131]. This transition imparts enhanced migratory and invasive capacity, survival advantage under stress, and increased resistance to apoptotic stimuli [39,131,132,133]. Notably, EMT is not always binary; tumor cells can exist in hybrid epithelial/mesenchymal states that further diversify the tumor landscape [134,135]. Such plasticity has been observed in multiple carcinoma and melanoma models, where EMT-generated intermediates exhibit stem-like properties, contribute to immune escape, and display altered responsiveness to conventional therapies [136,137]. By generating a spectrum of phenotypic states, EMT complements clonal evolution and CSC-driven mechanisms to reinforce tumor heterogeneity and therapeutic resistance [136,138].

The tumor stroma further exacerbates resistance through both biochemical and biomechanical means. While stroma in normal tissues may possess anti-tumor properties, tumor-associated stroma—comprising cancer-associated fibroblasts, ECM, endothelial cells, and immune cells—actively facilitates tumor initiation, invasion, and therapeutic resistance [139,140,141,142]. Bidirectional communication between tumor cells and the stroma triggers protective signaling cascades, contributing to what is termed Environment-Mediated Drug Resistance (EMDR) [143]. EMDR encompasses two major mechanisms: (1) Soluble factor-mediated resistance, involving cytokines, chemokines, and growth factors released by stromal elements that inhibit apoptosis in tumor cells. (2) Cell adhesion-mediated resistance, wherein tumor cell interaction with ECM components activates integrin-mediated signaling pathways that promote survival and reduce drug sensitivity [143,144,145]. In vivo studies have demonstrated that tumors exposed to repeated alkylating agent treatment in murine models developed significant resistance, which was not observed when the same cells were cultured in vitro, underscoring the essential role of stromal interactions in modulating therapeutic response [146].

In addition to biochemical crosstalk, the ECM in tumors is often excessively rigid and densely cross-linked, forming a formidable physical barrier that impedes drug diffusion [129]. Tumors can actively regulate ECM stiffness in response to metabolic and mechanical demands [147]. The increased matrix density and stiffness require therapeutic agents to traverse longer and more complex paths to reach their targets. Consequently, reduced drug penetration necessitates higher dosing, which may contribute to acquired resistance through adaptive cellular mechanisms, such as increased efflux pump expression and impaired apoptotic responses [148].

### 3.3. Impaired Fluid Flow and Abnormal Vasculature

The biophysical properties of fluid flow within tumors critically influence drug transport. Tumor interstitial fluid dynamics are governed by blood flow through disorganized vasculature, transvascular transport across endothelial barriers, and lymphatic drainage. In contrast to normal tissue, in which lymphatic vessels maintain fluid balance and facilitate macromolecule clearance, tumors exhibit deficient lymphatic drainage, leading to elevated IFP [149,150]. The movement of macromolecules from the plasma into the tumor interstitium is dictated by gradients in hydrostatic and oncotic pressure [151]. In one of the earliest direct measurements of tumor oncotic pressure, it was shown that while tumor tissues exhibit higher oncotic pressure than surrounding subcutaneous tissues, this pressure closely approximates that of plasma, resulting in a negligible pressure gradient [152]. This absence of gradient effectively inhibits transvascular convection and impedes the extravasation of large therapeutic molecules into the tumor parenchyma [113,152]. Furthermore, impaired lymphatic function disrupts convective flow within tumors.

In normal tissue, interstitial fluid movement is facilitated by a balance between vascular fluid input and lymphatic drainage [153]. In tumors, however, inadequate lymphatic clearance causes fluid retention, elevating IFP and suppressing convective transport [99,153]. Additionally, tumor-induced ECM remodeling and proliferative pressure can compress nearby vessels, further restricting blood flow. Consequently, drug distribution becomes reliant on diffusion alone, which is inefficient in the context of the dense ECM and spatial heterogeneity characteristic of solid tumors [99,154,155,156]. Together, these fluid-transport aberrations contribute to heterogeneous drug distribution, limited therapeutic penetration, and suboptimal treatment efficacy.

## 4. Limitations of Widely Used Tumor Models in Mimicking TME

In the early 1960s and 1970s, anticancer drug screening relied heavily on murine leukemia models, such as P388 and L1210, which implemented under the National Cancer Institute (NCI) [157,158]. Despite widespread use, these models yielded few successful clinical candidates, prompting the NCI to revise its screening strategy [159]. By the late 1980s, the NCI established a panel of 60 immortalized human tumor cell lines, known as the NCI-60, representing diverse tissue origins. These cell lines became the foundation of in vitro screening and continue to be used extensively for evaluating anticancer compounds [160,161,162,163].

The conventional drug screening workflow typically begins with testing compounds on tumor cells cultured as 2D monolayers, followed by evaluation in animal models [163,164,165]. Monolayer systems offer several advantages, including simplicity, high-throughput capability, reproducibility, and ease of interpretation [166,167]. However, despite their utility in mechanistic studies and target validation, these models fall short in predicting in vivo therapeutic efficacy. Numerous anticancer compounds that demonstrate robust activity in monolayer cultures fail to reproduce such outcomes in vivo [168,169,170,171]. A primary limitation of monolayer systems lies in their inability to recapitulate the complexity of the TME. Cells grown on flat plastic surfaces have unrestricted access to nutrients, oxygen, and therapeutic agents, unlike tumor cells in vivo that experience diffusion limitations due to disorganized vasculature and spatial gradients [172,173]. Moreover, monolayer cultures lack critical biophysical and biochemical features, such as cell–cell and cell–ECM interactions. These missing cues are known to regulate gene expression, drug responsiveness, and apoptotic resistance [172,174,175]. Gene expression studies comparing monolayer cultures to tumor tissues have demonstrated significant differences [176]. In a meta-analysis involving NCI-60 monolayers and clinical tumor samples, approximately 30% of ~7000 genes showed altered expression [176]. Genes associated with metabolism and macromolecular turnover were upregulated in monolayers, while those involved in adhesion and membrane signaling were downregulated [176]. In a separate study, chemoresistance in mammary tumor cells was shown to arise through integrin-mediated cell–ECM interactions that maintained polarity and suppressed apoptosis, underscoring the importance of ECM cues in drug response [177].

In vivo testing, while biologically more relevant, is not without limitations. Regulatory guidelines necessitate testing in both rodent and non-rodent species prior to clinical trials, with murine models favored for their low cost, rapid breeding, and amenability to genetic modification [178,179]. However, these models also present limitations. Key experimental parameters, such as animal sex, age, and handling stress are not consistently controlled across studies, contributing to variability [11,180]. Patient-derived xenografts (PDX), where human tumor tissues are implanted into immunodeficient mice, have been developed to increase clinical relevance. However, these models are expensive to establish and maintain, and they raise questions regarding tumor stage at engraftment—whether during early growth or after multiple mutations and metastatic progression—which directly influences therapeutic outcomes [180].

A critical biological limitation of in vivo models is that the TME in mice differs significantly from that in humans [179]. Even when human stromal cells are co-implanted with tumor cells, they are often replaced by murine stromal and immune cells over time [179]. This replacement compromises the tumor–stroma interactions that are essential for evaluating the therapeutic response in a human-like microenvironment [179,181]. Additionally, animal studies are subject to ethical and regulatory oversight, restricting the extent and scale of testing for multiple anticancer agents. Together, these limitations highlight the need for alternative models that are capable of preserving the architectural, cellular, and biochemical characteristics of human tumors while enabling reproducible and informative drug screening.

## 5. Spheroids as a More Physiologically Relevant TME Model

**Figure 2:**
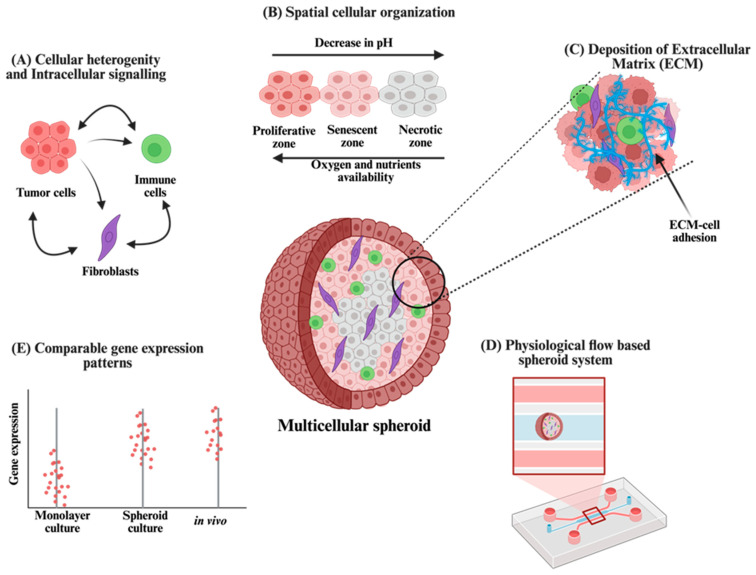
**Spheroid-based tumor models recapitulate key features of the in vivo tumor microenvironment.** (**A**) Cellular heterogeneity and intracellular signaling: Tumor spheroids incorporate diverse cell types, such as cancer cells, fibroblasts, and immune cells, enabling crosstalk via paracrine signaling pathways that reflect in vivo cellular interactions. (**B**) Spatial cellular organization: Gradients of proliferating, quiescent, and necrotic zones develop radially within the spheroid, mirroring the spatial compartmentalization observed in solid tumors. (**C**) Deposition of extracellular matrix (ECM): Stromal and tumor cells within spheroids secrete ECM components like collagen and fibronectin, contributing to matrix stiffness, structural integrity, and regulation of therapeutic penetration. (**D**) Physiological flow-based spheroid system: Integration of spheroids within microfluidic platforms facilitates the simulation of interstitial flow and dynamic nutrient exchange, enhancing physiological relevance and modeling of drug transport. (**E**) Comparable gene expression patterns: Schematic representation demonstrating how spheroids exhibit transcriptional profiles more similar to in vivo tumors than monolayer cultures, providing a more predictive model for evaluating therapeutic response and resistance.

To address the limitations associated with monolayer cultures and in vivo models, it is critical to adopt systems that replicate tumor structure and microenvironmental complexity. Among emerging in vitro models, multicellular tumor spheroids provide a platform that more accurately mimics the architecture, diffusion constraints, and cellular interactions present in solid tumors [112,182,183]. Spheroids are formed by the self-assembly of tumor cells into multicellular aggregates that generate physiological gradients in oxygen, nutrients, and waste products—features not present in monolayer cultures. These gradients typically emerge when spheroids exceed ~500 μm in diameter, with proliferative cells localized to the outer rim, quiescent cells residing in intermediate zones, and hypoxic or necrotic cores forming at the center due to limited diffusion [17,184,185]. As a result, spheroids allow for the evaluation of drug penetration, metabolic adaptation, and regional sensitivity within a tumor-like context [101,186]. Importantly, spheroid models can be co-cultured with stromal elements such as fibroblasts, endothelial cells, or immune cells. This permits the reconstitution of cell–cell and cell–ECM interactions that are essential for modeling tumor–stroma crosstalk, which governs processes such as survival signaling, drug resistance, and cell migration (Figure 2). These interactions are absent in monolayers but are necessary for recapitulating drug responses observed in vivo [101,186].

Moreover, several techniques have been optimized for evaluating spheroid models, including ATP-based viability assays, high-content imaging, and flow cytometry adapted for large particles. These methods enable quantification of treatment responses, spheroid integrity, and cellular composition over time. Importantly, spheroids can be produced in formats compatible with high-throughput screening, making them suitable for early-phase therapeutic testing.

This review will further discuss in detail the importance of including 3D spheroid models in routine screening of anticancer agents. Additionally, it will highlight established and emerging methods for evaluating drug response and other parameters in 3D cultures.

### 5.1. Internal Structure of Spheroids

Spheroids are characterized by a distinct spatial organization that reflects intratumoral gradients observed in solid tumors [101,184]. Cells in the outermost layer of the spheroid are typically highly proliferative due to their proximity to nutrients and oxygen. In contrast, cells located in the intermediate zone are often senescent, while those in the central core exist in a hypoxic state [101,184,186,187]. These patterns arise from restricted diffusion of oxygen and metabolites toward the spheroid interior, recapitulating key features of avascular tumor regions [113,187]. As previously described, cells residing in the hypoxic core undergo anaerobic metabolism, resulting in the accumulation of lactate and acidification of the extracellular environment. pH measurements within spheroid cores consistently range between 6.5 and 7.2, which closely mirrors the acidic pH observed in solid tumors [107,113]. This shift in pH can significantly influence therapeutic efficacy by altering drug stability, ionization, and intracellular uptake.

In addition to microenvironmental gradients, cells cultured in three-dimensional arrangements exhibit notable differences in morphology and physiology compared to cells grown in monolayers [188,189,190]. These differences are driven by both spatial and mechanical constraints inherent to 3D architecture [191]. The additional dimensionality impacts the orientation and clustering of cell-surface receptors, restricts cellular spreading, and alters cytoskeletal tension. As a result, intracellular signaling pathways and gene expression profiles are substantially modified [191,192].

It has been well established that gene expression patterns in spheroids more closely resemble those found in solid tumors than in monolayer cultures [112,193,194]. This shift is driven by the spatial organization, altered mechanical cues, and cell–matrix interactions present in three-dimensional environments. In one comparative study, transcriptomic analysis of melanoma cells cultured as monolayers, monolayers with ECM, and spheroids revealed that gene expression remained largely unchanged between the two monolayer conditions, while spheroid cultures exhibited substantial differences [194,195]. Over 100 transcripts were found to be upregulated and approximately 70 downregulated, with many of the altered genes associated with tumor progression and metastatic behavior [194]. A similar trend was observed in hepatocellular carcinoma models, where monolayers showed enrichment in structural gene expression, whereas spheroids upregulated genes related to metabolism and biosynthesis [196,197,198]. These findings have been corroborated across various tumor types and highlight that the transcriptional changes observed in spheroids are not limited to stress adaptation but support cellular phenotypes that facilitate survival, resistance, and disease progression.

Historically, such transcriptomic analyses have relied on bulk RNA extraction from dissociated spheroids, a method that loses spatial context and cell-specific resolution. As spheroid models become increasingly complex—incorporating multiple cell types and gradients of oxygen, nutrients, and drug exposure—there is a growing need for approaches that preserve spatial localization of gene expression. Emerging spatial transcriptomics platforms, such as MERSCOPE (Vizgen), FISSEQ, ExSeq, Slide-seq, and 10x Genomics Visium, now allow for high-resolution, multiplexed mapping of RNA molecules within intact 3D cultures [199,200,201]. These technologies provide valuable insights into how gene expression varies between proliferative rims and hypoxic cores, or among tumor and stromal populations within heterogeneous spheroids. Integrating such spatial omics approaches into spheroid analysis enables a more nuanced understanding of microenvironment-driven cellular states and can uncover emergent regulatory networks missed by bulk RNA-seq [201,202,203].

### 5.2. Cellular Heterogeneity and ECM Deposition in Spheroids

Multicellular tumor spheroids are appropriate systems for modeling tumor heterogeneity and microenvironmental complexity [204,205,206]. By incorporating stromal cell types, such as fibroblasts, endothelial cells, or immune cells, these models more closely replicate the cellular composition of in vivo tumors [182]. The ability to systematically vary the ratio of tumor to stromal cells enables the study of how microenvironmental components influence therapeutic response [207]. Experimental observations indicate that an increased proportion of stromal cells within spheroids reduces both drug penetration and treatment efficacy. In breast tumor models, spheroids generated with higher fibroblast content exhibited decreased diffusion of dextran dye and diminished cytotoxicity following doxorubicin exposure [208]. In pancreatic tumor models, spheroids formed using tumor cells, fibroblasts, and endothelial cells demonstrated greater drug resistance compared to spheroids lacking either stromal component [209]. These findings highlight the regulatory role of tumor–stroma interactions in modulating drug accessibility.

Spheroids also exhibit significantly higher levels of ECM deposition compared to monolayer cultures, particularly when stromal components are present [209,210]. The three-dimensional organization within spheroids creates a microenvironment where cells interact more extensively with matrix components, leading to enhanced expression and secretion of structural and regulatory ECM proteins. In colorectal tumor spheroids co-cultured with fibroblasts, expression of mesenchymal markers such as vimentin was markedly increased relative to tumor-only spheroids [40]. Moreover, the extent of ECM deposition scaled with fibroblast density, indicating that stromal cells actively contribute to matrix remodeling within the spheroid microenvironment [40]. Basement membrane extracts, such as Matrigel, have also been shown to enhance the structural integrity of spheroids [211]. Even small amounts of these ECM components can promote spheroid compactness by strengthening intercellular adhesion and tightening the interstitial space [40]. This increased compactness presents a substantial barrier to the diffusion of small molecules and macromolecular therapeutics, effectively limiting drug penetration and decreasing overall efficacy. In addition to acting as a physical barrier, the ECM can bind soluble factors and modulate integrin-mediated signaling, contributing to cell survival, resistance to apoptosis, and altered therapeutic response [212]. Beyond serving as a physical barrier, the incorporation of ECM during spheroid formation introduces a biomechanical stimulus that strongly influences cellular phenotype [213]. The matrix not only supports spheroid assembly but also imposes physical constraints that activate intracellular signaling cascades, including MAPK, PI3K/Akt, and YAP/TAZ pathways [214,215,216]. These mechanical cues have been shown to modulate transcriptional programs related to cell adhesion, survival, and drug resistance, leading to gene expression profiles that diverge significantly from those observed in ECM-free conditions [213,217,218]. Thus, ECM inclusion during spheroid formation does not merely stabilize structure—it functionally redefines cellular behavior in a manner more reflective of the in vivo tumor milieu.

In addition to modeling stromal influence and ECM organization, spheroid systems have also been utilized to study the behavior of CSCs, which are known to contribute to tumor heterogeneity, therapeutic resistance, and disease recurrence. Protocols have been established to isolate CSCs from patient-derived tumors or established cell lines using surface marker expression and flow cytometry. These CSC populations can then be cultured to generate spheroids that retain stem-like properties, including self-renewal and resistance to standard chemotherapeutics [219]. Comparative analyses have shown that spheroids derived from CSCs exhibit distinct morphologies and phenotypes relative to those formed from non-transformed epithelial cells. CSC-derived spheroids are typically larger, less organized, and display characteristics associated with increased malignancy, including loss of epithelial polarity, incomplete basement membrane coverage, and enhanced invasive behavior [220]. In contrast, spheroids formed from differentiated epithelial cells tend to exhibit well-defined architecture, strong cell–cell adhesion, and intact apical–basal polarity. These differences underscore the functional role of CSCs in driving aggressive tumor features and highlight the value of CSC-enriched spheroid models for studying metastasis and drug resistance mechanisms in a spatially relevant context [220].

### 5.3. Incorporating Physiological Flow into Spheroid Systems

Spheroids demonstrate growth kinetics similar to tumors during their avascular phase, characterized by rapid proliferation until they reach a diameter of approximately 600–800 µm, after which growth enters a plateau phase due to limitations in oxygen and nutrient diffusion. This plateau reflects the onset of metabolic stress and reduced proliferative capacity, closely mirroring the dormancy observed in early-stage solid tumors [48,182,221]. In vivo, this dormancy is typically overcome through angiogenesis, during which newly formed blood vessels introduce shear forces that sustain endothelial sprouts and support the delivery of therapeutic agents [49,222]. To simulate these physiological conditions, microfluidic platforms have been developed that integrate spheroid cultures with controlled flow [49,223]. These systems enable continuous delivery of oxygen, nutrients, and therapeutic agents while introducing shear stress and directional mass transport, thereby allowing the investigation of flow-regulated processes within a tumor-relevant architecture [224,225,226,227,228]. Initial studies examining the effects of fluid flow on cellular behavior utilized models in which endothelial cells were seeded into a central channel, flanked by a collagen I matrix containing embedded tumor cells [229]. When exposed to flow, these configurations exhibited increased expression of pro-angiogenic genes, indicating that mechanical forces can regulate angiogenic signaling and alter cellular responses to their environment [223].

Other microfluidic designs have been employed to investigate nanoparticle-based drug delivery in spheroid systems. One configuration included a perfused microchannel adjacent to a cavity containing multicellular tumor spheroids embedded in a polydimethylsiloxane matrix, representing a tumor surrounded by extracellular matrix [230]. Nanoparticles of varying sizes were introduced into the flow channel, and their transport was found to be size-dependent, with smaller particles penetrating more deeply into the spheroid [230]. Ligand functionalization of nanoparticles further enhanced their retention at the spheroid surface through receptor-mediated interactions [230]. When tested in vivo using the same tumor cells in xenograft models, comparable distribution patterns were observed, reinforcing the predictive utility of the in vitro platform [230]. Recent advances have led to the development of tri-channel systems that structurally model key features of the tumor microenvironment [231]. In one such platform, endothelial cells were cultured along the lumen of the first microchannel to simulate vascular interfaces [231]. The adjacent channel was polymerized with ECM components to serve as a diffusive barrier, while the third microchannel was engineered with U-shaped microwells designed to spatially localize mammary gland spheroids. This configuration enabled direct visualization of nanoparticle movement across endothelial and matrix compartments and into the spheroid body, allowing quantitative analysis of spatiotemporal drug transport under defined flow conditions.

## 6. Strategies to Characterize Structural and Functional Features of Spheroids

While the development of spheroid models has significantly advanced the ability to mimic the structural and functional features of solid tumors, it is equally essential to characterize their properties both prior to and following therapeutic intervention. Accurate analysis of spheroid morphology, viability, metabolic state, and response to treatment provides critical insight into their biological relevance and translational value. These analyses are necessary to ensure that observed treatment outcomes are reflective of physiologically relevant behavior, and to dissect the mechanisms underlying therapeutic resistance or efficacy.

**Figure 3:**
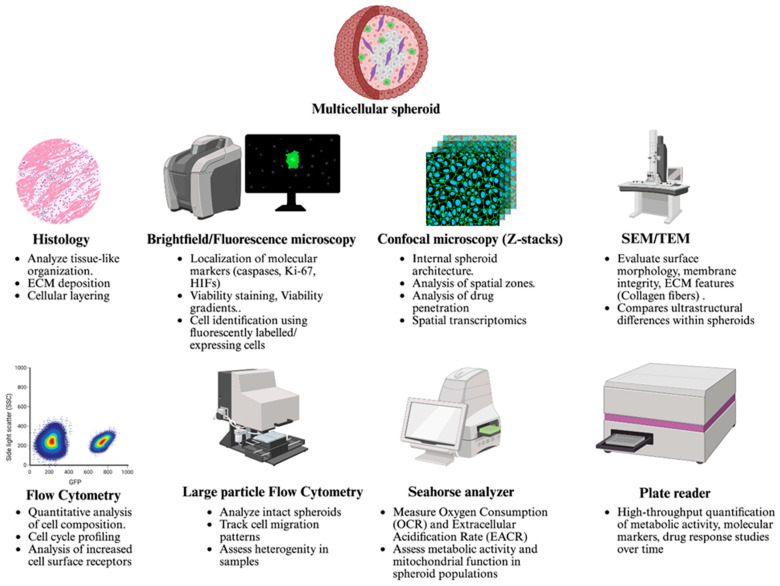
**Representative analytical platforms and their uses to characterize spheroid structure and function.**

In response to these needs, a range of experimental platforms and analytical tools have been developed to assess spheroid features at morphological, molecular, and functional levels (Figure 3). Advances in high-content imaging, optical sectioning, live–dead staining, and multiplexed viability assays have enabled increasingly detailed evaluation of drug distribution, penetration, and cytotoxicity within spheroid models. Furthermore, newer platforms allow for the integration of dynamic measurements, such as oxygen gradients, cell cycle progression, and metabolic flux, offering a deeper understanding of spatial and temporal treatment effects.

The following sections will examine key techniques that have been employed to quantitatively and qualitatively assess spheroid characteristics. These include methods for evaluating spheroid size and structure, cellular viability and apoptosis, proliferative index, and drug penetration. Together, these approaches provide a framework for interpreting therapeutic outcomes within the context of the complex tumor-like architecture present in spheroid systems.

### 6.1. Imaging Techniques to Study Growth and Organization

A range of microscopic techniques has been employed to evaluate spheroid growth, morphology, and internal cellular organization [14,182,232]. These include brightfield, widefield fluorescence, and confocal and electron microscopy, with each offering unique advantages depending on the parameter being assessed. The most direct approach for monitoring spheroid expansion is brightfield imaging using an optical microscope equipped with a calibrated camera [233,234]. Spheroid diameter can be measured either in real-time using imaging software with built-in measurement tools or retrospectively through analysis with ImageJ, an open-source image processing program developed by the National Institutes of Health. Differences in light transmission through hypoxic versus proliferative zones of the spheroid can be visualized under brightfield, although this method lacks the resolution and specificity required to characterize cell states across distinct layers.

To overcome these limitations, fluorescence microscopy is widely used in conjunction with molecular and metabolic dyes to assess cellular status. Fluorescently labeled antibodies targeting markers such as caspase-3, Ki-67, and hypoxia-inducible factors, or exogenous hypoxia indicators such as pimonidazole and EF5, allow visualization of apoptotic, proliferating, or hypoxic regions, respectively [235,236]. In parallel, live–dead cell discrimination can be achieved using dyes such as Calcein AM, fluorescein diacetate, or acridine orange to stain viable cells, while ethidium homodimer, ethidium bromide, or propidium iodide are used to identify nonviable populations [112,237]. When applied in combination, these stains enable quantitative and spatial evaluation of viability across the spheroid. Beyond general viability assessments, the spatial localization and modulation of specific molecular markers provide critical insights into cellular function, stress response, and therapeutic efficacy within spheroids. Key markers that can be assessed include nuclear proteins such as phospho-histone H3 (a mitotic marker), γH2AX (a DNA damage response marker), and cleaved PARP or active caspase-3 (apoptosis indicators) [238]. Membrane and cytoplasmic proteins like EGFR, HER2, and β-catenin are also commonly evaluated to assess pathway activation, receptor internalization, or EMT transitions [239]. These markers can be detected via immunofluorescence and quantitatively imaged across the spheroid using confocal microscopy or high-content imaging platforms. Spatial tracking of marker expression—particularly in response to therapeutic agents—can reveal distinct biological responses within proliferative outer zones, hypoxic intermediate layers, or necrotic cores [240,241,242]. By comparing marker distribution before and after treatment, researchers can evaluate drug-induced alterations in cell state, pathway activation, and resistance phenotypes in a depth-resolved, physiologically relevant context.

In addition to viability and marker localization, fluorescence-based techniques have been extended to histological assessments. Stains such as hematoxylin and eosin (H&E), toluidine blue, and Masson’s trichrome have been used to evaluate tissue-like organization within spheroids and provide insight into ECM composition [186,243]. However, conventional wide-field fluorescence microscopy is limited in its ability to resolve fluorescence signals within thick or optically dense spheroids as it collects out-of-focus light from the entire specimen and projects it onto a single focal plane. This results in reduced contrast and loss of spatial resolution, particularly for large spheroids [244]. Two primary strategies are commonly employed to address this [245]. The first involves physically sectioning the spheroid into thin slices using cryo-sectioning or microtomy, followed by fluorescence imaging of individual sections. While this method improves depth resolution, it may compromise structural fidelity due to freezing-induced ice crystal formation or mechanical disruption during sectioning [246,247]. The second strategy involves optical sectioning through confocal laser scanning microscopy, which acquires fluorescence from successive focal planes along the Z-axis to generate high-resolution Z-stacks of the spheroid’s internal structure [186,248]. Nevertheless, both techniques face limitations when imaging spheroids exceeding several hundred micrometers in diameter due to scattering and absorption of excitation and emission light within intact tissue [249]. To overcome this, spheroid clearing methods—such as lipid removal using chemical agents—have been developed to enhance light penetration by reducing refractive index mismatches and optical opacity [250,251,252]. When combined with confocal or light-sheet microscopy, cleared spheroids allow for deep imaging of entire 3D structures while preserving fluorescence labeling. This facilitates more accurate spatial analysis of fluorescently labeled cell populations, surface proteins, or reporter constructs throughout the spheroid volume. More advanced imaging platforms have emerged to overcome residual limitations in depth and phototoxicity. Techniques such as multiphoton and light sheet fluorescence microscopy allow for deeper penetration, reduced photobleaching, and higher imaging speed. These methods have been particularly useful in capturing dynamic biological processes and resolving complex cellular arrangements within intact spheroids [253,254,255].

Electron microscopy, including both scanning (SEM) and transmission (TEM) modalities, provides ultra-high-resolution imaging and remains a valuable tool for evaluating cellular morphology, surface topology, and intercellular organization across spheroid layers [245,256]. Although more resource-intensive, electron microscopy enables detailed visualization of membrane structures, organelle integrity, and ECM [257]. For instance, comparative SEM studies of drug-sensitive and drug-resistant tumor spheroids have revealed distinct surface morphologies following treatment, including loss of membrane integrity in drug-sensitive cells. In other cases, SEM imaging has revealed the presence of extracellular fiber-like structures on the spheroid surface, later confirmed to be collagen, underscoring the technique’s utility in visualizing matrix remodeling and stromal features [258]. More recently, second harmonic generation (SHG) microscopy has emerged as a label-free imaging modality capable of visualizing collagen-rich structures within intact spheroids [259,260]. SHG leverages the non-centrosymmetric nature of fibrillar collagens to generate signal without the need for fluorescent tags, enabling deep-tissue imaging with minimal photodamage [259,261]. When combined with optical clearing techniques, SHG microscopy allows high-resolution, three-dimensional mapping of ECM architecture, offering insights into spatial collagen organization, remodeling dynamics, and stromal–tumor interactions under physiologically relevant conditions [261]. Taken together, these imaging platforms provide critical insight into the spatial and functional organization of spheroids. By enabling real-time, depth-resolved, and ultrastructural characterization, microscopy plays an essential role in assessing spheroid formation, heterogeneity, and response to therapeutic intervention.

### 6.2. Identifying Distinct Cell Populations in Spheroids

Multicellular tumor spheroids reflect the cellular heterogeneity of in vivo tumors, and this heterogeneity is one of the primary reasons for their increasing adoption in preclinical modeling. However, capturing the diversity of cell populations and identifying functional differences such as proliferative capacity or hypoxic status within the spheroid requires optimized analytical strategies. One of the most effective approaches involves the use of cell lines genetically engineered to express distinct, non-toxic fluorescent proteins. These cell lines are commercially available through specialized suppliers and allow for precise identification and spatial tracking of individual cell types. In cases where pre-labeled cell lines are not available, stable or transient transfection can be employed to introduce fluorescent proteins into specific cell populations prior to spheroid formation [262,263,264]. Once labeled, the populations can be distinguished using fluorescence microscopy or flow cytometry, enabling high-resolution characterization of spheroid composition. When genetic labeling is not feasible due to cell-type-specific limitations, an alternative strategy involves pre-labeling cells with membrane-permeable fluorescent dyes prior to aggregation [265,266]. For instance, in co-culture models containing epithelial and stromal components, fibroblasts have been selectively labeled with membrane-integrating dyes to facilitate post-formation discrimination [266]. However, this method presents notable limitations. First, dense and compact spheroids may impede uniform dye penetration, resulting in incomplete labeling. Second, over time, the dye may diffuse into adjacent non-labeled cells, leading to inaccurate identification and signal overlap. These challenges must be carefully considered when selecting labeling strategies for spheroid-based analyses.

Beyond population tracking, assessment of cell cycle status provides further insight into functional heterogeneity within spheroids [267,268]. Fluorescent nucleotide analogs such as 5-bromo-2′-deoxyuridine (BrdU) or 5-ethynyl-2′-deoxyuridine (EdU) are commonly employed to detect cells in the S phase of the cell cycle by incorporating into newly synthesized DNA [267]. These probes enable distinction between proliferating and senescent populations, particularly when comparing spheroid cultures to monolayer systems. For example, in comparative analyses, cells in monolayers often exhibit a higher proportion of S-phase activity than those within spheroids, reflecting the diffusion-limited and quiescent microenvironment within the spheroid core [267]. Additional fluorescent reporters, such as Fluorescent Ubiquitination-based Cell Cycle Indicator systems, allow for the discrimination of G1 and S-M phase populations using spectrally distinct signals [268,269]. These tools are particularly valuable in dynamic live-cell tracking and in understanding treatment-induced cell cycle arrest. However, care must be taken when applying these dyes in combination with fluorescent drugs or pre-labeled cells, as spectral overlap can compromise data accuracy. To mitigate such interference, several biotechnology platforms have developed optimized fluorochrome panels with minimal emission overlap, designed to enable multiplexed detection across multiple laser lines.

More recently, fluorescence lifetime imaging microscopy (FLIM) has also emerged as a refined method to assess proliferation in 3D models with improved specificity and reduced signal interference [270,271]. A notable application involves quantifying the quenching of Hoechst 33342 fluorescence lifetime upon BrdU incorporation, enabling detection of S-phase cells using a single nuclear dye [270,272]. This approach bypasses intensity-based limitations and eliminates the need for multiple fluorophores, making it particularly suitable for live-cell, high-content imaging of spheroids and organoids [273]. Importantly, FLIM-BrdU has been successfully applied to both monolayer and 3D models, providing real-time, multiparametric insight into cell cycle dynamics and drug responses in intact, physiologically relevant systems [273].

### 6.3. Evaluating Cellular Migration in Spheroids

Understanding cellular migration within spheroids is essential for modeling tissue remodeling, stromal activation, and tumor progression. In vivo, stromal cells such as fibroblasts and endothelial cells do not remain statically embedded but actively reposition themselves in response to gradients in oxygen, nutrients, and soluble factors [274,275]. This repositioning plays a critical role in shaping ECM organization, promoting angiogenesis, and facilitating immune modulation—each of which contributes to therapy resistance and tumor evolution [21,79,275]. Capturing such spatiotemporal dynamics in vitro is therefore necessary for establishing spheroid models that accurately reflect microenvironmental behavior observed in solid tumors. Migration analysis can also provide mechanistic insight into how different cell types interact under spatial constraint and biochemical stress—features often absent from conventional monolayer systems.

**Figure 4:**
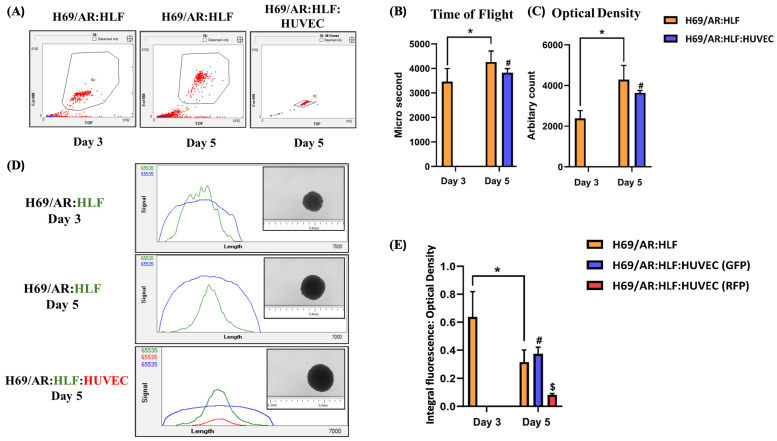
**Large particle flow cytometer (COPAS Vision) analysis of multicellular spheroids.** (**A**) COPAS Vision dot plots representing diameter measurement Time of Flight (TOF) vs. Optical density (EXT) (**B**) TOF measurements (diameter) of spheroids in microseconds (**C**) Arbitrary count of Optical density (EXT) (**D**) Representative profiler plots of signal intensity across individual spheroids Optical density (blue), green fluorescence (green), and red fluorescence (red) for each plot, inset shows corresponding monochrome image of spheroid analyzed. (**E**) Integral fluorescence to Optical Density ratio—for day 3 and day 5 old H69/AR:HLF co-culture and day 5 old H69/AR:HLF:HUVEC triple co-culture spheroids. Data represents mean ± standard deviation (n > 200). Student *t*-test * *p* < 0.01 vs. day 3 H69/AR:HLF, # *p* < 0.01 vs. day5 H69/AR:HLF spheroid. $ *p* < day 5 H69/AR:HLF spheroid.

A practical strategy for studying intra-spheroidal cell migration involves fluorescent labeling of distinct cell types prior to spheroid formation. Fluorescent markers allow for real-time tracking of individual populations without disturbing the spheroid architecture. When paired with high-content fluorescence microscopy or large-particle flow cytometry, these approaches enable spatial resolution of cell distribution patterns at various time points [276]. Large-particle flow cytometry is particularly advantageous, as it permits the analysis of intact spheroids based on physical and optical properties while preserving information about cellular localization and spheroid integrity [276,277]. Unlike conventional flow cytometry, which requires enzymatic dissociation of spheroids into single-cell suspensions, large-particle flow cytometry enables the analysis of intact spheroids while preserving their physical structure and spatial fluorescence distribution. In the representative study shown in Figure 4, co-culture and triple co-culture spheroids were generated using Adriamycin-resistant lung carcinoma cells (H69/AR), human lung fibroblasts (HLFs), and human umbilical vein endothelial cells (HUVECs) [278]. Prior to spheroid formation, HLFs were labeled with green fluorescent protein (GFP), while HUVECs expressed red fluorescent protein (RFP), facilitating spatial analysis of each population within the 3D structure [278]. Spheroid size and compactness were assessed using two parameters: Time of Flight (TOF) and Optical Density (OD). As shown in the bar plots, both parameters increased significantly from day 3 to day 5 in both co-culture and triple co-culture conditions, indicating progressive growth and compaction of the spheroids. Importantly, the profiler plots (Figure 4C) depict signal intensity profiles across individual spheroids, where the blue signal corresponds to total optical density (representing the spheroid mass), and the green signal represents the distribution of HLFs. On day 3, the green fluorescence signal from HLFs was broadly distributed and overlapped with the blue signal, suggesting that fibroblasts were evenly dispersed throughout the spheroid [278]. By day 5, however, the green fluorescence became concentrated toward the center, with diminished signal at the periphery, indicating active migration of HLFs toward the spheroid core [278]. This central accumulation of fibroblasts is consistent with known in vivo behavior, where stromal cells such as fibroblasts migrate toward hypoxic regions to participate in extracellular matrix remodeling and survival signaling [279,280,281,282]. Notably, the red fluorescence signal from HUVECs was also observed to cluster near the green-labeled fibroblasts, suggesting preferential spatial association [278]. This behavior is consistent with the known paracrine interactions in which fibroblasts secrete pro-angiogenic factors such as VEGF and Fibroblast Growth Factor 2 (FGF2), which facilitate endothelial cell growth and organization [283,284]. The spatial proximity of HUVECs to HLFs supports the idea of stromal-guided endothelial localization and provides further evidence of biologically relevant cell–cell interactions within these 3D systems [285]. In addition to hypoxia, fibroblasts respond to a variety of chemotactic signals such as TGF-β, PDGF, and CXCL12, which are differentially distributed within tumors and further drive their directed migration toward tumor cores [286]. Similar behavior has been observed in 3D in vitro systems, where fibroblasts embedded in hydrogel or co-culture matrices exhibit directed migration toward spheroid interiors under hypoxic stress [287]. In melanoma spheroids, for example, fibroblasts have been shown to accumulate in low-oxygen regions and upregulate genes associated with ECM production and wound response [288]. Such findings highlight that stromal cell migration in 3D systems is not merely a passive process, but is instead a dynamic adaptation to biochemical and metabolic gradients. Beyond fibroblasts, similar strategies have been used in immune-tumor spheroids to investigate how macrophages or T cells migrate in response to tumor-derived chemokines, influencing immune suppression, matrix remodeling, or drug resistance [289].

Thus, quantitative tracking of these processes is increasingly facilitated by integrated platforms combining large-particle flow cytometry, fluorescent protein tagging, and live imaging. These tools provide high-resolution, population-scale insight into redistribution dynamics, enabling deeper analysis of how physical constraints and biochemical gradients drive spatial organization. Ultimately, the ability to monitor intra-spheroidal migration in a temporally and spatially resolved manner adds critical dimensionality to the functional evaluation of spheroid models. These migratory behaviors—often missed by conventional endpoint assays—offer a dynamic view of how cell–cell and cell–matrix interactions evolve over time and influence therapeutic outcomes under physiologically relevant conditions.

### 6.4. Assessing Drug Penetration in Spheroids

Assessing the penetration of therapeutic agents or delivery systems within a spheroid is critical for evaluating in vivo efficacy and determining appropriate dosing strategies. Penetration is governed by the physicochemical characteristics of the drug or particle—including size, surface charge, shape, lipophilicity, and acid dissociation constant—as well as by the structural and biochemical properties of the spheroid, such as ECM density, extracellular pH gradients, and compactness of the cellular arrangement [99,206,290].

One of the most accessible methods for studying intratumoral distribution is the use of intrinsically fluorescent compounds. Several chemotherapeutic agents, such as doxorubicin and epirubicin, exhibit inherent fluorescence, enabling direct visualization of their spatial distribution within spheroids using fluorescence or confocal microscopy [291,292]. Penetration depth can be quantified by imaging serial sections of the spheroid or by generating optical Z-stacks with confocal microscopy [246,247]. When drugs or particles lack inherent fluorescence, they can be conjugated to fluorescent dyes prior to administration, allowing similar assessment of diffusion and accumulation patterns [293,294]. Confocal microscopy has been widely used to evaluate penetration profiles and is also capable of providing quantitative insights [294,295]. For instance, the relative binding of a molecule can be estimated by calculating the percentage of total fluorescence intensity confined to the outer 20 µm rim of the spheroid, which typically corresponds to the outer two to three cell layers. Similarly, overall affinity can be inferred by summing the fluorescence intensity across all sections [293]. As an advanced example of this type of analysis, the Determination of Nanoparticle Uptake in Tumor Spheroids platform has been developed to spatially and temporally map nanoparticle distribution in intact spheroids [294]. This approach enables quantitative assessment of uptake kinetics, depth-dependent accumulation, and cell-type-specific differences in nanoparticle internalization, providing high-resolution insight into how physical properties and spheroid architecture affect transport behavior [294]. Despite these advantages, confocal microscopy has limitations, including photobleaching due to broad excitation light exposure and limited penetration depth caused by photon scattering in dense spheroid tissue. These constraints can reduce imaging accuracy, particularly in larger or more optically opaque spheroids [296]. To address these limitations, two-photon excitation microscopy has emerged as a non-destructive alternative. This technique allows for deeper tissue imaging by restricting excitation to the focal plane, thereby minimizing photodamage and photobleaching. The longer excitation wavelengths used in two-photon microscopy also reduce scattering, enabling improved visualization of inner spheroid regions and providing enhanced depth resolution for analyzing drug distribution [297,298].

Flow cytometry offers a more sensitive and quantitative platform for evaluating drug uptake at the single-cell level. However, it requires enzymatic or mechanical dissociation of the spheroid, which disrupts spatial information and complicates localization of specific cell populations within the spheroid architecture. Traditionally, this has limited its application in assessing penetration depth. Nevertheless, approaches have been developed to infer spatial gradients through differential staining patterns [299]. For instance, fluorescent DNA-binding dyes can be applied under controlled incubation times to generate a concentration gradient, whereby cells at the periphery exhibit stronger staining than those deeper within the spheroid. When coupled with fluorescently labeled therapeutic agents, these gradients can be visualized as bivariate fluorescence distributions, providing insights into relative penetration and uptake across different formulations [300]. By integrating fluorescence imaging with high-resolution cytometric and spectroscopic tools, researchers can extract both spatial and quantitative data regarding the behavior of therapeutic agents in three-dimensional tumor models. These analyses contribute significantly to understanding how drug properties and spheroid microarchitecture influence therapeutic accessibility and effectiveness, informing the design and optimization of delivery strategies for solid tumors.

### 6.5. Evaluating Therapeutic Efficacy in Spheroids

A fundamental parameter in anticancer drug evaluation is the ability of the compound to induce cytotoxic effects within the tumor microenvironment. In spheroid-based assays, drug efficacy is often assessed by comparing changes in spheroid size over time relative to untreated controls [301,302,303]. This can be performed non-invasively using brightfield microscopy to monitor spheroid diameter as a function of time. While this method is straightforward and permits real-time assessment, certain limitations exist. Imaging directly in the treatment well can compromise resolution due to medium interference or debris. To mitigate this, spheroids are often cultured in ultra-low attachment plates or transferred to clean imaging wells prior to measurement to ensure consistent visualization [304,305]. Despite its utility, microscopy-based growth inhibition analysis does not provide mechanistic insight or detailed viability information. Prolonged treatments may result in partial disintegration of the spheroid structure, complicating morphological evaluation and making longitudinal comparisons unreliable [206]. Consequently, additional biochemical and cell viability assays are frequently employed to quantify therapeutic effects more accurately.

Conventional viability assays, such as MTT and MTS, which rely on the enzymatic reduction of tetrazolium salts to formazan by metabolically active cells, are commonly used in monolayer systems but require optimization for three-dimensional cultures due to diffusion limitations [306]. Reagent penetration can be insufficient in compact spheroids, necessitating extended incubation times or modifications to assay protocols [306]. For example, extended reagent exposure or parallel quantification using monolayer standards have been implemented to ensure more accurate estimation of viable cell numbers in 3D systems [186,305]. To address these challenges, several viability assays specifically formulated for three-dimensional cultures have been developed. Commercial kits, such as CellTiter-Glo^®^ 3D and Cultrex^®^ 3D viability assays, are designed to enhance lysis efficiency and signal uniformity in spheroids [15,307]. Additional markers of cellular integrity, including lactate dehydrogenase release, caspase activation, and changes in mitochondrial membrane potential, have also been adapted to evaluate apoptotic and necrotic responses within 3D tumor models [186,308,309,310].

Beyond these biochemical assays, efforts have been made to employ advanced spectroscopic techniques to capture spatial and compositional changes associated with treatment response [311,312,313]. Techniques such as proton-induced X-ray emission, X-ray fluorescence microscopy, and Fourier-transform infrared spectroscopy have been used to map elemental and biochemical distributions across spheroid layers [313]. For example, spectroscopic analyses have revealed increased levels of sulfur and potassium in spheroid cores, attributed to stress-induced thiol upregulation under hypoxic conditions. In contrast, peripheral regions often display elevated protein (β-sheet) and lipid content, consistent with higher metabolic activity. These methods enable non-destructive, label-free quantification of biomolecular changes and have been employed to evaluate treatment-induced shifts in metabolic profiles following exposure to platinum-based anticancer agents [313].

## 7. Conclusions

The clinical success of anticancer therapeutics for solid tumors remains limited by poor drug accumulation within the tumor mass and insufficient retention at the site of action. Effective delivery strategies must overcome a range of physiological barriers to ensure adequate drug concentrations in the tumor core, particularly in the context of heterogeneous microenvironments. Consequently, the design and optimization of drug delivery systems (DDS) that enhance intratumoral penetration and retention continue to be a central focus in oncology research. Despite encouraging outcomes in traditional in vitro models, many DDS platforms fail to replicate their efficacy in preclinical animal models. This translational gap is often attributed to the widespread reliance on monolayer cell cultures, which do not adequately represent the structural and functional complexity of in vivo tumors. Two-dimensional systems lack key physiological features, such as spatial cell–cell and cell–matrix interactions, ECM-mediated transport resistance, cellular heterogeneity, oxygen and nutrient gradients, and dynamic mass transport—all of which influence therapeutic response.

Spheroid models address many of these shortcomings by capturing essential aspects of solid tumor biology, including three-dimensional architecture, proliferation gradients, cell-type diversity, and matrix-rich microenvironments. Furthermore, their tunability, compatibility with high-content imaging and biochemical assays, and capacity for integration into microfluidic systems make them a versatile and scalable tool for drug development. By more closely reflecting in vivo tumor behavior, spheroids enable more discriminating assessment of therapeutic efficacy, drug penetration, and mechanistic pathways, improving the predictive accuracy of early-stage screening. Integrating spheroid models into routine preclinical workflows not only enhances the physiological relevance of in vitro testing but also has the potential to reduce dependence on animal models, thereby aligning scientific rigor with ethical responsibility. As the field moves toward more sophisticated and predictive platforms, the widespread adoption of spheroid-based assays will be critical for bridging the gap between bench research and clinical translation in cancer therapy.

## Data Availability

No new data were created or analyzed in this study.

## Abbreviations

BrdU	5-Bromo-2′-Deoxyuridine
CAF	Cancer-Associated Fibroblast
CSC	Cancer Stem Cell
DDS	Drug Delivery System
ECM	Extracellular Matrix
EMDR	Environment-Mediated Drug Resistance
EMT	Epithelial-to-Mesenchymal Transition
FAK	Focal Adhesion Kinase
FFA	Free Fatty Acid
FLIM	Fluorescence Lifetime Imaging Microscopy
FGF2	Fibroblast Growth Factor 2
H69/AR	Adriamycin-Resistant Lung Carcinoma Cells
HLF	Human Lung Fibroblast
HUVEC	Human Umbilical Vein Endothelial Cell
IFP	Interstitial Fluid Pressure
NCI	National Cancer Institute
NK	Natural Killer (Cell)
MAPK	Mitogen-activated Protein Kinase
O.D.	Optical Density
PI3K	Phosphoinositide 3-kinase
ROS	Reactive Oxygen Species
SEM	Scanning Electron Microscopy
SHG	Second Harmonic Generation
TAM	Tumor-Associated Macrophage
TEM	Transmission Electron Microscopy
TME	Tumor Microenvironment
T.O.F	Time-of-Flight
VEGF	Vascular Endothelial Growth Factor
YAP/TAZ	Yes-associated protein/Transcriptional coactivator with PDZ-binding motif
